# Assessing the Quality and Reliability of Videos Related to Fibromyalgia on TikTok: A Comprehensive Analysis

**DOI:** 10.7759/cureus.64704

**Published:** 2024-07-17

**Authors:** Ahmed N Canatan

**Affiliations:** 1 School of Medicine, Marmara University, Istanbul, TUR

**Keywords:** patient education, health information, social media, tiktok, fibromyalgia

## Abstract

Introduction

Fibromyalgia, characterized by chronic musculoskeletal pain and associated symptoms, poses significant challenges in diagnosis and management. While social media platforms like TikTok have emerged as popular sources of health information, their variable content quality necessitates critical evaluation. This study aimed to assess the quality and reliability of TikTok videos related to fibromyalgia, thereby enhancing the understanding of their impact on patient education and self-management.

Methods

A cross-sectional observational study was conducted in June 2024, which analyzed 150 TikTok videos using search terms like "Fibromyalgia", "Fibromyalgia Symptoms", and "Fibromyalgia Treatment". Videos were evaluated for inclusion based on relevance and language (English), by employing the Global Quality Scale (GQS) and Quality Criteria for Consumer Health Information (DISCERN) score for assessment. Statistical analysis was performed by using IBM SPSS Statistics v21.0 (IBM Corp., Armonk, NY). The Kruskall-Wallis test was employed, and a p-value less than 0.05 was deemed statistically significant.

Results

Of the 150 videos initially reviewed, 96 (64%) met the inclusion criteria. Content categories included disease description (34, 35.42%), symptoms (81, 84.38%), management (64, 66.67%), and personal experiences (63, 65.63%). The videos were uploaded by doctors (8, 8.33%), patients (63, 65.63%), healthcare workers ( 7, 7.29%), and others (18, 18.75%). Mean GQS scores varied significantly by uploader type: doctors (4.63 ± 0.52), healthcare workers (3.43 ± 0.79), patients (2.37 ± 0.81), and others (2.11 ± 0.47) (p<0.001). DISCERN scores followed a similar trend: doctors (3.88 ± 0.64), healthcare workers (2.14 ± 1.46), patients (1.08 ± 0.27), and others (1.61 ± 0.50) (p<0.001).

Conclusions

TikTok serves as a pivotal platform for fibromyalgia-related discourse, predominantly shaped by patient-generated content. However, even though it provides insights into symptoms and management strategies, gaps exist in comprehensive medical guidance and preventive measures. The study underscores the critical role of healthcare professionals in enhancing content reliability and educational value on social media. Future research should explore cultural and linguistic diversity to broaden the accessibility and relevance of health information on platforms like TikTok.

## Introduction

Fibromyalgia is a chronic condition characterized by widespread musculoskeletal pain, fatigue, and tenderness in localized areas [1]. It is a complex disorder that affects the nervous system, resulting in amplified pain signals [1]. Fibromyalgia affects approximately 5% of the global population, with a higher incidence in women compared to men [1]. The condition typically manifests between the ages of 30 and 35 years [1]. Many individuals diagnosed with fibromyalgia experience symptoms such as sleep disturbances, cognitive difficulties, and mood disorders, significantly impacting their quality of life [2].

Diagnosing fibromyalgia is challenging, requiring the extensive experience and discretion of physicians [3]. This evaluation often involves discussing the patient's symptoms, medical history, and lifestyle factors to rule out other potential causes of chronic pain [4]. The current fibromyalgia criteria are useful for classifying patients in surveys, research, and clinical settings but fall short in terms of capturing the full complexity of the condition [3]. Important factors like invalidation, psychosocial influences, and varied disease expression are often overlooked [3]. Thus, evaluating fibromyalgia patients should involve a thorough and nuanced approach, viewing fibromyalgia holistically rather than relying solely on specific criteria or scores [3].

Managing fibromyalgia typically requires a multifaceted approach [5]. This may include medication to reduce pain and improve sleep, physical therapy to increase strength and flexibility, and psychological support to address associated mental health issues [5]. Lifestyle modifications such as regular exercise, stress management techniques, and dietary adjustments are also important components of a comprehensive treatment plan [5,6]. Patient education and self-management strategies play a vital role in the long-term management of fibromyalgia [5,7]. Patients are encouraged to stay informed about their condition and actively participate in their treatment plans [7]. The internet, specifically TikTok, has become a popular resource for individuals seeking information about fibromyalgia [8]. However, the quality and reliability of the information available on these platforms can vary significantly, potentially leading to misinformation and inadequate self-care practices [8].

The primary aim of this study is to evaluate the information on fibromyalgia available on TikTok; the study endeavors to assess the quality and reliability of these videos by using the Global Quality Scale (GQS) and Quality Criteria for Consumer Health Information (DISCERN) scores. We aim to provide a clearer understanding of the current landscape of fibromyalgia-related content on TikTok and its potential impact on patient education and self-management.

## Materials and methods

A cross-sectional observational study was carried out in June 2024 to determine the quality and reliability of uploaded information on fibromyalgia on TikTok. A total of 150 videos were evaluated by using the search terms "Fibromyalgia", "Fibromyalgia Symptoms", "Fibromyalgia Treatment", "Living with Fibromyalgia", “Fibromyalgia Awareness” and "Fibromyalgia Pain". It is important to note that the data collection through video evaluation was conducted in the Republic of Türkiye, as TikTok's algorithm tends to prioritize displaying geographically local content to viewers initially. Videos in English of any duration and relevant to the topic of fibromyalgia were included in the study. Videos not related to fibromyalgia were considered irrelevant content and excluded from the final analysis. Duplicate videos, as well as videos not in the English language, were also excluded from the final statistical analysis.

**Figure 1:**
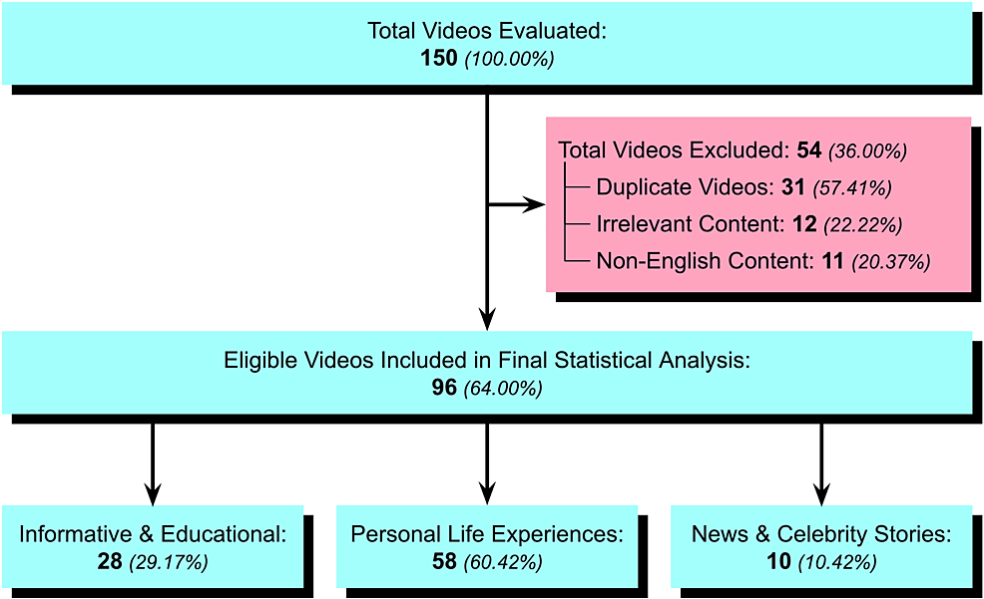
Flowchart depicting study selection

Out of 150 videos evaluated, 96 (64%) were eligible to be included in the study based on the inclusion criteria. A total of 54 (36%) videos were excluded from the study for either having inappropriate content, inappropriate language, or being a duplicate. Among the videos included in the final statistical analysis, 28 (29.17%) were informative and educational on the topic of fibromyalgia, 58 (60.42%) involved personal life experiences related to living with fibromyalgia, and 10 (10.42%) were from news organizations discussing celebrities suffering from fibromyalgia (Figure 1).

The videos were analyzed for initial metrics (number of likes, comments, and followers), the content related to fibromyalgia (covering disease description, symptoms, management, awareness, epidemiology, causes, diagnosis, treatments, rehabilitation, support groups, personal stories, and promotional content), the type of uploader (doctor, patient, healthcare worker, and others), and the overall quality and reliability of the information presented. 

The GQS and DISCERN scores were used to evaluate the quality and reliability of the videos. The GQS is a metric used to evaluate the quality of health-related content in online videos, articles, and other informational resources. It offers a standardized way to assess the overall quality and reliability of the information, using a scale from 1 to 5. A score of 1 signifies very poor quality, with content that is inaccurate, misleading, unprofessional, unclear, and lacking in educational value. A score of 2 indicates fair quality, where the content has some merit but remains subpar, with information that may be incomplete or somewhat inaccurate, presentation that may be unclear, and limited educational usefulness. A score of 3 represents moderate quality, with generally accurate information that may lack depth or detail, adequate presentation, and some educational value. A score of 4 is given to good quality content, which is accurate, reasonably comprehensive, clear, professional, and offers significant educational value. A score of 5 denotes excellent quality, with highly accurate, comprehensive, and detailed information, presented in an engaging, clear, and professional manner, providing high educational value. The GQS is used by laymen, researchers, and healthcare professionals to evaluate the reliability and educational usefulness of health information across various media [9].

The DISCERN score is a tool designed to assess the quality and reliability of video or written consumer health information. It involves responding to five questions with either "yes" or "no," where each "yes" signifies a positive attribute and is worth 1 point. The first question inquires if the video clearly states its objectives, with a "yes" indicating that the purpose is explicitly defined and easy to understand. The second question evaluates the reliability of the information sources, and a "yes" suggests that references and evidence are well-documented and trustworthy. The third question assesses whether the publication is impartial and unbiased; a "yes" shows that it presents various viewpoints without favoring a particular treatment. The fourth question looks at whether additional sources are listed for patients, with a "yes" indicating that supplementary references are provided for further information. Finally, the fifth question asks if areas of uncertainty are stated, with a "yes" showing that the video acknowledges and discusses the limitations or gaps in the information presented. The total number of "yes" answers is added up to give a DISCERN score out of 5, indicating the overall quality of the health information in the video [10]. 

Statistical analysis of the eligible 96 videos that had passed the inclusion-exclusion criteria mentioned above was done by using IBM SPSS Statistics v21.0 (IBM Corp., Armonk, NY). The Kruskal-Wallis test was used to identify any statistical significance between two or more groups of an independent variable on a continuous dependent variable (e.g., type of uploader versus GQS or DISCERN score). Quantitative data are presented as mean ± standard deviation (SD). Statistical analysis was conducted at a confidence level of 95%, and a p-value below 0.05 was considered statistically significant. Figures and tables were prepared using Google Slides and Google Sheets, respectively.

## Results

Table 1 presents the characteristics of the TikTok videos evaluated in this study on fibromyalgia-related content. It includes the total number of audience reached by the videos, which reflects a substantial engagement with a cumulative number of likes reaching 1,811,760, comments totaling 77,430, and a combined number of followers amounting to 16,009,722. The table also categorizes the type of uploader for these videos, revealing that the majority were uploaded by patients (63, 65.63%). These metrics provide insights into the reach and popularity of fibromyalgia-related content on TikTok, illustrating the platform's potential as a significant source of information and community engagement for individuals affected by chronic illnesses like fibromyalgia.

**Table 1:** Characteristics of videos (n=96)

Characteristic	N (%)
Total number of audience reached by the videos	
Number of likes	1,811,760
Number of comments	77,430
Number of followers	16,009,722
Type of uploader	
Doctor	8 (8.33%)
Patient	63 (65.63%)
Healthcare worker	7 (7.29%)
Others	18 (18.75%)

Table 2 provides a breakdown of the criteria covered in TikTok videos related to fibromyalgia, highlighting the frequency of specific content categories. The study covered a total of 13 distinct topics related to fibromyalgia. The most frequently discussed topics were awareness and recognition of fibromyalgia, which was covered in 88 (91.67%) posts, and symptoms of fibromyalgia, mentioned in 81 (84.38%) posts. Management of fibromyalgia and personal experiences of living with fibromyalgia were discussed in more than half of the posts, accounting for 64 (66.67%) and 63 (65.63%) posts, respectively. Other topics such as the description of fibromyalgia (34, 35.42%), prevention of fibromyalgia flare-ups (29, 30.21%), rehabilitation for fibromyalgia (28, 29.17%), support groups for fibromyalgia (27, 28.13%), treatment of fibromyalgia (23, 23.96%) and cause of fibromyalgia (21, 21.88%) were addressed to varying extents. The topics mentioned the least were related to the prevalence and epidemiology of fibromyalgia (8, 8.33%), diagnosis of fibromyalgia (7, 7.29%), and promotional content for a pharmaceutical company or medication related to fibromyalgia (2, 2.08%). Table 2 provides an overview of the thematic focus of fibromyalgia-related content on TikTok, shedding light on the diversity and emphasis of information available to viewers on this platform.

**Table 2:** Type of information shared in the videos

Criteria	N (%)
Awareness and recognition of fibromyalgia	88 (91.67%)
Symptoms of fibromyalgia	81 (84.38%)
Management of fibromyalgia	64 (66.67%)
Personal experiences of living with fibromyalgia	63 (65.63%)
Description of fibromyalgia	34 (35.42%)
Prevention of fibromyalgia flare-ups	29 (30.21%)
Rehabilitation for fibromyalgia	28 (29.17%)
Support groups, counseling, and therapy for fibromyalgia	27 (28.13%)
Treatment of fibromyalgia	23 (23.96%)
Cause of fibromyalgia	21 (21.88%)
Prevalence and epidemiology of fibromyalgia	8 (8.33%)
Diagnosis of fibromyalgia	7 (7.29%)
Promotional content for a pharmaceutical company or medication	2 (2.08%)

Table 3 presents the GQS and DISCERN scores for TikTok videos related to fibromyalgia, categorized by the type of uploader. The mean GQS scores indicate varying levels of content quality, with videos uploaded by doctors receiving the highest score (4.63 ± 0.52), followed by healthcare workers (3.43 ± 0.79), patients (2.37 ± 0.81), and others (2.11 ± 0.47). Similarly, DISCERN scores, which assess the reliability of health information, also show doctors' videos with the highest score (3.88 ± 0.64), followed by healthcare workers (2.14 ± 1.46), patients (1.08 ± 0.27), and others (1.61 ± 0.50). The Kruskal-Wallis test results indicate statistically significant differences (p<0.001) in both GQS and DISCERN scores across the different types of uploaders, emphasizing the influence of uploader type on the quality and reliability of fibromyalgia-related content on TikTok. These findings underscore the importance of considering the source of information when assessing the educational value and trustworthiness of health-related content on social media platforms.

**Table 3:** Assessment of quality and reliability of videos based on the type of uploader P-value <0.05 is deemed statistically significant DISCERN: Quality Criteria for Consumer Health Information; GQS: Global Quality Scale; SD: standard deviation

Type of uploader	N (%)	GQS score, mean ± SD	DISCERN score, mean ± SD
Doctor	8 (8.33%)	4.63 ± 0.52	3.88 ± 0.64
Patient	63 (65.63%)	2.37 ± 0.81	1.08 ± 0.27
Healthcare worker	7 (7.29%)	3.43 ± 0.79	2.14 ± 1.46
Others	18 (18.75%)	2.11 ± 0.47	1.61 ± 0.50
P-value (Kruskal-Wallis test)		<0.001	<0.001

## Discussion

Recent years have underscored the pivotal role of social media in disseminating public health information and preventive health advice [11]. Since 2013, the emergence of diverse social media applications has led to significant benefits, particularly for individuals seeking health information or coping with illnesses [11]. Governmental use of social media, especially during disease outbreaks, has proven advantageous for the broader community [12]. Recent studies have expanded the focus beyond patients, encompassing the general public and health professionals, to understand how different groups utilize social media for health-related purposes [12]. These explorations correspond with our analysis of TikTok videos about fibromyalgia, revealing a significant audience comprising creators and consumers - ranging from doctors to predominantly patients and others unrelated to healthcare - with the quality and reliability of the content being posted on health topics such as fibromyalgia varying considerably. This underscores the need for regular assessment and regulation to ensure the reliability of health-related content on social media platforms.

The 96 TikTok videos analyzed in this study garnered substantial attention, reflected by 1,811,760 likes, 77,430 comments, and 16,009,722 followers, indicating a broad reach. A similar study found a significant reliance on social media for health information during the pandemic [13]. In their study, over three-quarters of respondents reported using social media at least "a little," and more than half read COVID-19-related information weekly, indicating the heavy reliance on social media for health-related topics [13]. In our analysis of TikTok videos on fibromyalgia, we found that the majority of content creators were patients (63, 65.63%), followed by others (18, 18.75%), doctors (8, 8.33%), and healthcare workers (7, 7.29%). These findings parallel those from other studies which highlight the significant engagement of patients in disseminating health information via social media [13]. However, the variation in the type of uploader and the substantial audience engagement underscores the critical need to assess and ensure the quality and reliability of the information being shared.

In our study, doctors and healthcare workers accounted for the smallest proportion of TikTok videos related to fibromyalgia, comprising (8, 8.33%) and (7, 7.29%) of the videos, respectively. This corresponds with the conclusions of another study that highlighted ongoing uncertainty about the roles and responsibilities of physicians who share medical content on social media platforms [14]. Additionally, it was noted that only a minority of providers seemed to be utilizing the platform to its fullest extent [14]. Our analysis suggests that patients are the primary content creators in this context, similar to other studies that indicate that patients frequently share health-related experiences and information on social media [13]. This emphasizes the ongoing need for healthcare professionals to engage more actively on these platforms to ensure the accuracy and reliability of the information being disseminated.

Our study analyzed TikTok videos on fibromyalgia, covering disease description, symptoms, management, awareness, prevalence, causes, diagnosis, prevention of flare-ups, treatment, rehabilitation, support groups, personal experiences, and pharmaceutical promotions. This illustrates the extensive range of information circulating online and accessed by patients about fibromyalgia and other chronic illnesses, highlighting the critical role of clear healthcare communication from medical professionals. A poll conducted by Mediabistro revealed that over half of participants had changed their treatment or preventive practices based on information they found online, and more than a fourth were likely to change their minds after seeking online information [15]. Our findings highlight the need for accurate and reliable health information on social media platforms, particularly given the high percentage of personal experience content. Online health information has a significant impact on public behavior, reinforcing the need for credible sources to guide patients' decisions and actions regarding their health management [15].

In our study, only 29 (30.21%) of the TikTok videos focused on the prevention of fibromyalgia flare-ups. This is similar to the findings of another study regarding obesity, where only a quarter of the posts they analyzed addressed obesity prevention, indicating that preventive messages are a common but not predominant focus in health-related social media content [16]. Public health authorities can leverage social media platforms to effectively disseminate targeted health messages and increase public awareness. However, more research is needed to determine how these platforms can enhance health literacy and promote healthy behaviors across different cultural contexts.

Social media users engage in sharing, discussing, and observing each other's health journeys, a phenomenon also evident in our investigation where 63 (65.63%) of the TikTok videos focused on personal experiences of living with fibromyalgia [17]. Patients often find solace and motivation in sharing their own stories, which can aid in coping with their condition and achieving health goals. This sharing also benefits other users by connecting them with individuals who have similar experiences, facilitating peer support, lifestyle advice, and exploration of treatment options. This aligns with research indicating that social media platforms serve as valuable spaces for individuals to exchange health-related narratives and foster supportive communities [17]. Further exploration into how these platforms influence health behaviors and patient empowerment could provide valuable insights into enhancing patient care and support strategies.

In our study, TikTok videos uploaded by doctors exhibited significantly higher GQS scores compared to those uploaded by healthcare workers, patients, and other contributors. This finding resonates with the findings of another study focusing on pediatric scoliosis where videos uploaded by patients received notably lower quality and reliability scores [18]. Additionally, a related analysis on obesity found that posts uploaded by doctors tended to have a higher percentage of accurate information, further supporting our observation of doctors' contributions on social media platforms [16]. We also found that posts uploaded by doctors received significantly higher DISCERN scores compared to those uploaded by healthcare workers, patients, and other contributors.

Our findings align with the findings of another study that looked at videos related to patient education for the meniscus, where videos uploaded by doctors also showed the highest JAMA scores [19]. The study emphasized significant differences in reliability and educational quality based on the source of video upload, reinforcing our observation of doctors' contributions being perceived as more reliable across different health-related content platforms [19]. Conversely, videos uploaded by patients in both studies tended to have lower ratings, indicating potential challenges in ensuring the accuracy and comprehensiveness of health information shared by non-professional sources [19]. These findings underscore the critical role of healthcare professionals in maintaining the quality and reliability of health-related information on social media, highlighting opportunities for enhancing educational content and user engagement strategies to benefit patient education and support.

In our analysis of TikTok videos on fibromyalgia, while we focused on the quality and reliability of content uploaded by different types of creators, we acknowledge the broader issue of misinformation in health communication on social media. Social media platforms have become primary vehicles for the proliferation of health misinformation [20]. The prevalence of misinformation on social media platforms is alarming, with studies revealing that up to 87% of certain health-related posts contain inaccurate information [20]. Influencers and content creators sometimes endorse dietary supplements without robust scientific backing, influenced by partnerships with manufacturing companies [20]. This phenomenon is concerning as it can lead to misleading health claims and misguided consumer choices, despite the limited efficacy associated with these products [20]. Addressing this challenge requires collaborative efforts among healthcare professionals, platform providers, and regulatory bodies to ensure that accurate and trustworthy health information prevails on these influential platforms.

Limitations

This study on assessing the quality and reliability of TikTok videos related to fibromyalgia has several limitations that warrant consideration. Firstly, the decision to include only English-language videos excluded potentially valuable content from other languages, possibly overlooking cultural nuances and diverse perspectives that could enhance understanding of fibromyalgia. Furthermore, the search strategy relied on a limited set of six common keywords, potentially missing videos tagged with specific phrases used exclusively by individuals with fibromyalgia. Additionally, the subjective nature of video quality assessment, conducted by a single observer using the GQS and DISCERN instrument, introduces a bias that could be mitigated by multiple observers scoring videos.

Moreover, due to TikTok's privacy policies, the country of residence of the uploaders is not publicly disclosed, making it impossible for us to include such an important metric in our analysis. While it may be feasible to infer the country of residence for some uploaders by searching for their private information online, this approach would be inappropriate. Future studies could potentially address this limitation by collaborating directly with TikTok and other social media platforms on which they are conducting their study, to obtain anonymized location data and ensure user privacy while enabling more comprehensive analyses. Lastly, the dynamic nature of TikTok means video content can change rapidly, affecting the relevance and timeliness of study findings over time. These limitations underscore the need for careful interpretation of the study's findings and the importance of exploring areas for future research on how social media platforms can educate patients about their chronic illnesses.

## Conclusions

Our comprehensive analysis of TikTok videos related to fibromyalgia underscores the significant role of social media in shaping public perceptions and disseminating health information. Our findings reveal a diverse landscape of content creators, predominantly patients, contributing to a substantial engagement on the platform. However, the variability in content quality and reliability emphasizes the urgent need for ongoing scrutiny and regulation to ensure accurate health information dissemination. Unlike platforms where medical experts dominate, TikTok reflects a scenario where patients share personal experiences, representing a unique avenue for peer support and community building. Nevertheless, given the influence of online health information on individual behaviors, it remains crucial for healthcare professionals to actively engage with these platforms, thereby enhancing the credibility and educational value of health-related content. By fostering a collaborative effort between healthcare providers and social media users, we can cultivate a more informed and health-conscious community.
